# Monitoring an Emergent Pathogen at Low Incidence in Wastewater Using qPCR: Mpox in Switzerland

**DOI:** 10.1007/s12560-024-09603-5

**Published:** 2024-05-23

**Authors:** Timothy R. Julian, Alexander J. Devaux, Laura Brülisauer, Sheena Conforti, Johannes C. Rusch, Charles Gan, Claudia Bagutti, Tanja Stadler, Tamar Kohn, Christoph Ort

**Affiliations:** 1 Eawag, Swiss Federal Institute of Aquatic Science and Technology, Ueberlandstrasse 133, 8600 Dübendorf, Switzerland; 2 Swiss Tropical and Public Health Institute, 4123 Allschwil, Switzerland; 3 University of Basel, 4001 Basel, Switzerland; 4 Department of Biosystems Science and Engineering, ETH Zurich, 4009 Basel, Switzerland; 5 State Laboratory of Basel-Stadt, Basel, Switzerland; 6 Swiss Institute of Bioinformatics, 1015 Lausanne, Switzerland; 7 Laboratory of Environmental Virology, School of Architecture, Civil and Environmental Engineering, (ENAC), École Polytechnique Fédérale de Lausanne, Lausanne, Switzerland; 8 Present Address: Institute of Integrative Biology, Department of Environmental Systems Science, ETH Zurich, Zurich, Switzerland

**Keywords:** Wastewater-based epidemiology, Mpox, qPCR, Limit of detection

## Abstract

Wastewater-based epidemiology offers a complementary approach to clinical case-based surveillance of emergent diseases and can help identify regions with infected people to prioritize clinical surveillance strategies. However, tracking emergent diseases in wastewater requires reliance on novel testing assays with uncertain sensitivity and specificity. Limited pathogen shedding may cause detection to be below the limit of quantification or bordering the limit of detection. Here, we investigated how the definition of limit of detection for quantitative polymerase chain reaction (qPCR) impacts epidemiological insights during an mpox outbreak in Switzerland. 365 wastewater samples from three wastewater treatment plants in Switzerland from 9 March through 31 October 2022 were analyzed for mpox DNA using qPCR. We detected mpox DNA in 22% (79 of 365) wastewater samples based on a liberal definition of qPCR detection as any exponentially increasing fluorescence above the threshold. Based on a more restrictive definition as the lowest concentration at which there is 95% likelihood of detection, detection was 1% (5 of 365). The liberal definition shows high specificity (90%) and accuracy (78%), but moderate sensitivity (64%) when benchmarked against available clinical case reporting, which contrasts with higher specificity (98%) but lower sensitivity (10%) and accuracy (56%) of the 95% likelihood definition. Wastewater-based epidemiology applied to an emergent pathogen will require optimizing public health trade-offs between reporting data with high degrees of uncertainty and delaying communication and associated action. Information sharing with relevant public health stakeholders could couple early results with clear descriptions of uncertainty.

**Impact Statement:** When a novel pathogen threatens to enter a community, wastewater-based epidemiology offers an opportunity to track its emergence and spread. However, rapid deployment of methods for to detect a novel pathogen may rely on assays with uncertain sensitivity and specificity. Benchmarking the detection of mpox DNA in Swiss wastewaters with reported clinical cases in 2022, we demonstrate how definitions of detection of a qPCR assay influence epidemiological insights from wastewater. The results highlight the need for information sharing between public health stakeholders that couple early insights from wastewater with descriptions of methodological uncertainty to optimize public health actions.

**Supplementary Information:**

The online version contains supplementary material available at 10.1007/s12560-024-09603-5.

## Introduction

Wastewater-based epidemiology offers a promising approach to detect the emergence and spread of novel pathogens within communities (Boehm et al., 2023; Heijnen & Medema, 2011; Kazama et al., 2016; McCall et al., 2020; Peccia et al., 2020; Wolfe et al., 2021). Although the focus of many wastewater-based surveillance programs is to quantify disease dynamics of endemic or epidemic pathogens in a community, one potential application is to identify the introduction and spread of a novel pathogen within communities. Early information on the geographic spread of pathogens can help us to inform and prioritize public health interventions, such as preparing hospitals, or motivating changes to testing strategies or community-level interventions. As shown with SARS-CoV-2, wastewater-based detection may demonstrate wider geographic dispersion of the disease than the first clinical case reports alone (Cariti et al., 2022). This use of wastewater was also applied to other pathogens circulating in communities at low incidence, such as the detection of poliovirus in Israel, the UK, and the USA, which prompted corresponding public health interventions to reduce cases and associated paralysis (Manor et al., 1999).^1^

An outstanding question on the application of wastewater for pathogen detection at low prevalence is method sensitivity. For SARS-CoV-2, sensitivity estimates vary, with a reported estimate that wastewater can be used to reliably detect as few as 10 reported cases per 100,000 people served by the catchment (Bagutti et al., 2022; Hewitt et al., 2022; Medema et al., 2020). These estimates are influenced by a series of factors, including the strength of clinical cases identification and the sensitivity of detection methods. Pathogen-specific attributes that influence sensitivity include the load of pathogens shed into wastewater per infection, the prevalence of shedding among infected people, and the persistence of pathogen nucleic acids during transport in sewer networks (Hata and Honda, 2020).

Rapid implementation of wastewater-based surveillance to track a novel pathogen at low disease prevalence will initially produce data characterized by at least some uncertainty. Because assay detection methods may be novel, there may be insufficient data on assay sensitivity and specificity. Compounding this, results from analysis of wastewater may result in: 1) positive signals at or below standard definitions of the limit of quantification (LOQ) or detection (LOD); 2) detection of pathogens with inconsistent replicate results, potentially due to low concentrations or sample inhibition; and 3) false positive detection due to non-specific amplification or laboratory contamination. False positives are particularly concerning, as incorrect interpretation may lead to unnecessary and costly interventions, or inaccurate epidemiological insights. There is a movement in molecular and environmental biology toward increased rigor in the development, use, and reporting of data obtained from PCR-based methods (Borchardt et al., 2021; Bustin et al., 2009). This movement is an attempt to bolster comparability and reproducibility toward better informed public health actions (Borchardt et al., 2021).

Uncertainty in the implementation of wastewater-based surveillance to track diseases at low disease prevalence is confounded by imperfect clinical case data. Assessing utility of wastewater-based surveillance, including assay sensitivity and specificity, depends on comparison to clinical data. However, the clinical data likely fail to capture all cases or may report occurrence of cases temporally misaligned with the timing within which pathogens are shed into wastewater. In particular, clinical cases represent only a portion of infections, and the data quality depends on many factors including the frequency and severity of symptoms, health-seeking behaviors of infected people, and the strength of testing. During SARS-CoV-2 emergence in Geneva, Switzerland, for example, confirmed cases represented less than 10% of those estimated in a seroprevalence study (Stringhini et al., 2020).

Although a cautious approach in interpreting uncertain data is prudent, there is an associated cost to the resulting delay in relaying information to relevant public health agencies. In scenarios where there is uncertain data, additional analyses are advocated for, including the use of multiple distinct targets, sequencing samples to confirm pathogen identity, and attempting to further concentrate samples to increase detectability. There is an ethical imperative to ensuring that quality assurance and quality control measures are followed, but also that results are timely communicated (Hrudey et al., 2021). Delays in public health actions allow continued transmission, resulting in increased infections and limiting opportunities to prevent epidemics (Zou et al., 2022). There are therefore trade-offs between communicating uncertain results and delaying communication to ensure quality.

The introduction and spread of mpox in Europe in the spring and summer of 2022 offered an opportunity to benchmark sensitivity of wastewater against clinical case reporting for a pathogen of low incidence (Tiwari et al., 2023). In Switzerland, the first clinical case was reported on 19 May.^2^ Reported cases rose rapidly, reaching 419 total cases in Switzerland by 22 August. The rapid increase in cases in Switzerland raised concerns of an epidemic, with substantial uncertainty on the extent of the disease transmission or anticipated trajectory. Within the scope of COVID-19 surveillance, raw wastewater samples were analyzed to quantify SARS-CoV-2 RNA during the mpox outbreak. A subset of these samples were leveraged to detect mpox DNA using a quantitative polymerase chain reaction (qPCR).

In this study, we estimate the sensitivity of wastewater-based mpox detection by assessing the relationship between infrequent, low detection of mpox DNA, and reported clinical cases during the outbreak in Switzerland from 19 May 2022 through 9 October 2022. The decision to analyze the samples for mpox DNA was driven by reports of shedding of mpox DNA in urine, feces, and skin scabs (Pieró-Mestres et al., 2022), which could all act as sources of DNA into the wastewater. Furthermore, multiple laboratories reported successful detection of mpox DNA in wastewater (Jonge et al., 2022; La Rosa et al., 2023; Sharkey et al., 2022; Wolfe et al., 2023; Wurtzer et al., 2022). Through this integrated analysis, we evaluate the application of uncertain data obtained from wastewater for detection of an emergent pathogen at low incident.

## Methods

### Reported Mpox Cases

Mpox cases were reported to the Swiss Federal Office of Public Health starting on 21 of May^2^. To preserve case anonymity, case data were made available for this study aggregated by week and canton. For two of the wastewater treatment plants (WWTP), the population served represents approximately 30% of the cantonal population (WWTP Lugano serves 124,000 people of the 353,000 people in the canton of Ticino; and WWTP Werdhölzli serves 471,000 people of the 1.5 million people in the canton of Zurich). WWTP Aïre serves approximately 90% of the population (454,000 of the 499,000 people in the canton of Geneva). The cantonal case data may therefore overestimate the number of the cases within the catchment. For analyses, we use cantonal case data and do not adjust for the proportion of the population served because of the potential urban clustering of mpox cases and the primarily urban coverage of the three monitored wastewater treatment plant catchments.^3^

### Wastewater Sampling

365 wastewater samples from three wastewater treatment plants (WWTP Aïre, Geneva; CDA Lugano, Lugano; ARA Werdhölzli, Zurich) from 9 March through 31 October 2022 were analyzed for mpox DNA.

Within the context of surveillance for mpox clinical cases, samples were processed every one to two days between June 9 and October 31. To inform the specificity of the assay, one sample per week (Wednesdays) from March and April 2022 was analyzed, which represents a period before any clinical cases of mpox were reported and could act as negative controls. These samples were analyzed using archived DNA/RNA stored at −80 °C.

### DNA Extraction

Total nucleic acid (TNA) was extracted from 40 ml of raw influent using a modified version of the Wizard® Enviro Total Nucleic Acid Kit (Promega Corporation, Madison, USA). DNA/RNA was eluted in 80 μl, and further purified using a OneStep PCR Inhibitor Removal Kits (Zymo Research, Irvine, USA) to reduce assay inhibition. TNA extracts were processed either after storage at −80 °C for up to three months (March through mid-July samples) or processed immediately (mid-July through October samples).

### Quantitative PCR

Samples were analyzed on the Quantstudio 3 Real-Time PCR System (Applied Biosystems, Massachusetts, USA) with the QuantStudio Design and Analysis Software V2 (version 2.6.0) using the mpox assay (primers, probes, and cycling conditions) specified by the Pan American Health Organization (Table 1), with primers and probe synthesized by Microsynth AG (Balgach, Switzerland).^4^ The mastermix used was GoTaq (Promega, Wisconsin, USA) with a 20 μl reaction volume, of which 5 μl was template. On July 27, a reference dye, Carboxy-X-Rhodiamine (CXR), was added to better normalize the fluorescent signal and improve thresholding. For most samples (359, or 98%), qPCR reactions were performed in duplicate. However, 6 samples were performed in singlet due to insufficient volume of DNA/RNA extract.

**Table 1:** Primers and probe used for detection of mpox, from Pan American Health Organization (2022)

G2R_G forward primer	5′-GGAAAATGTAAAGACAACGAATACAG-3′
G2R_G reverse primer	5′-GCTATCACATAATCTGGAAGCGTA-3′
G2R_G probe	5′FAM-AAGCCGTAATCTATGTTGTCTATCGTGTCC-3′BHQ1

Laboratory guidelines for the detection and diagnosis of monkeypox virus infection

Standard curves were generated using commercially available plasmids (TibMolBiol CN: 30-7580-01) and included replicates of ten-fold dilutions from 5 × 10^6^ to 50 copies per reaction. No template controls (NTCs) were included in every run. To generate comparable standard curves across experiments, the thresholds were set such that the mean Ct for replicate samples of 400 copies per reaction standard was 32.5. Standards were then pooled to generate a universal standard curve to estimate concentrations of positive samples (Figure S1). Standard curves were pooled to mediate the impact of inter-experimental variation in standard curve performance (due to, for example, pipetting variations in master mix and standard curves) on estimated concentrations. Conceptually, pooling of standard curves should better align Cts and associated concentration estimates for samples processed during distinct qPCR runs. To determine sensitivity of the assay, additional standard curves were created with dilutions down to 0.5 genome copy equivalents (gce) per reaction.

### Calculation of LOD and LOQ

LOD was calculated using three approaches: 1) the lowest concentration at which 95% of samples were detected, 2) dose–response modeling as specified by Klymus et al. (2020), and 3) detection of exponentially-increasing fluorescence above the threshold prior to the terminal 45th PCR cycle. The latter approach defines a LOD based on distinguishing a signal from the NTCs. All data analyzed were quantified based on the pooled standard curves.

LOQ estimates were found using a polynomial extrapolation modeling approach (parameters set to “best”, or the model with the lowest residual standard error) specified by Klymus et al. (2020) (using data quantified based on the pooled standard curves Klymus et al., 2020). Quantification of mpox gce was determined from the pooled standard curves. Measured mpox gce were converted to wastewater concentrations (gce per ml) and then multiplied by the wastewater treatment plants daily flow rate to calculate load (gce per day).

## Statistical Analysis

All statistics were performed with R (version 4.1.2, R Foundation for Statistical Computing, Vienna, Austria). All qPCR data were analyzed as detect/non-detect data aggregated by calendar week to align with clinical cases that were similarly reported. Logistic regression was used to determine the likelihood of detecting mpox DNA in a catchment for a given calendar week as a function of the number of reported mpox clinical cases in the corresponding canton.

## Results

### Reported Cases

**Fig. 1:**
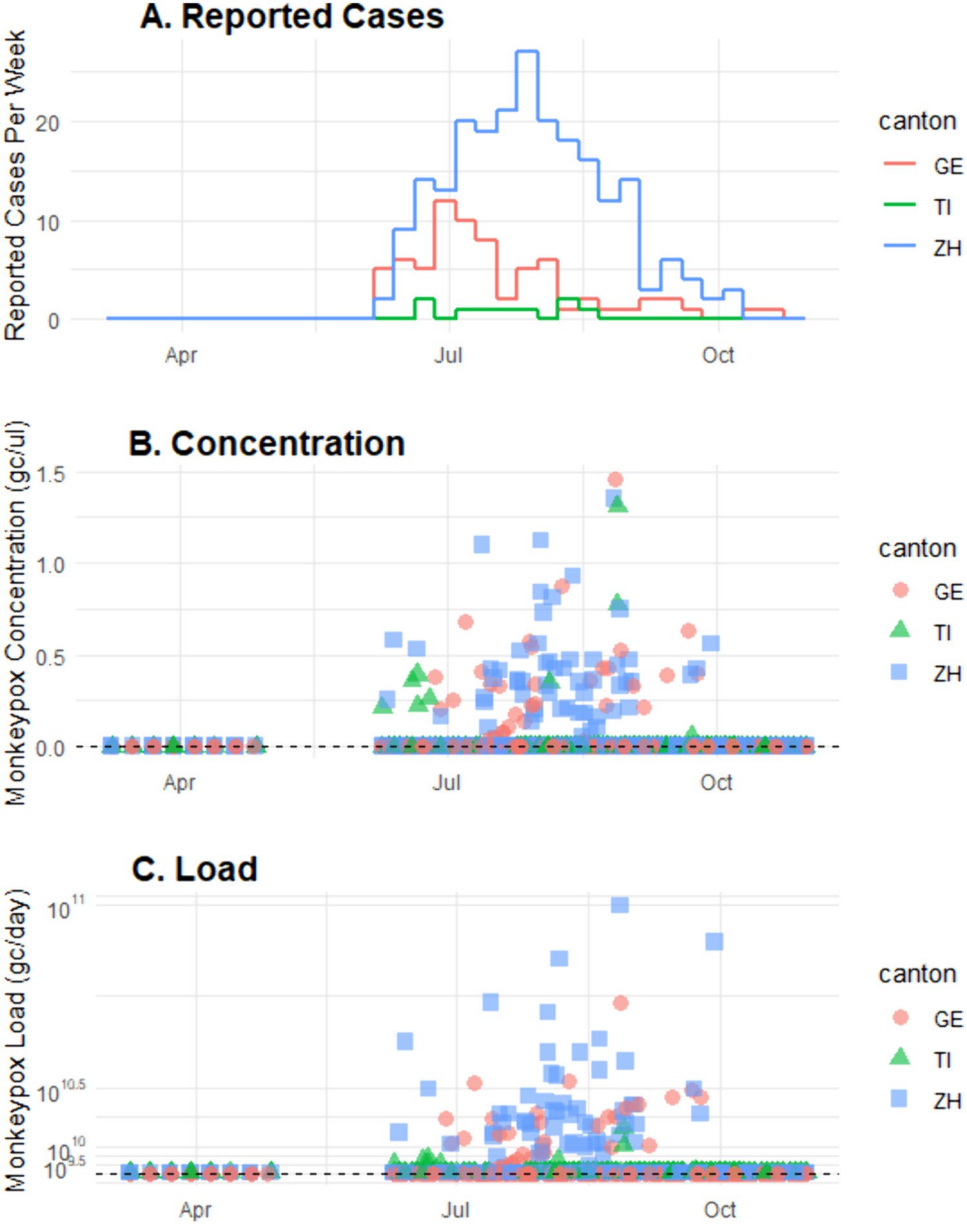
**A** Reported weekly mpox cases in cantons of Geneva (GE), Ticino (TI), and Zurich (ZH) from March through October 2022 and corresponding **B** maximum observed concentrations (gce per ml wastewater) and **C** maximum observed loads (gce per day) of mpox DNA measured in wastewater treatment plants within the catchment. Notably, concentrations and loads were determined based on extrapolation of the standard curve below the determined LOQ

During the period of the study (16 May until 23 October 2022), the Swiss Federal Office of Public Health reported 74 cases in Geneva, 8 cases in Ticino, and 227 cases in Zurich cantons (Fig. 1A). The corresponding incidence rates, reported here in cases per 100,000 people in each canton over the 161 days monitored, were 0.15 for Geneva, 0.02 for Ticino, and 0.15 for Zurich.

### Quantitative PCR Performance

As determined from pooling the standard curves across all experiments consisting of 302 standards, the slope (−3.46) of the qPCR assays corresponds to an amplification efficiency of 94.5%, with an intercept of 39.4. The pooled standard curve has an *R*^2^ of 99.9%. The LOQ of the assay is 135 copies per reaction (Ct of 34.5), based on polynomial extraction modeling (Klymus et al., 2020). The standard with fifty copies per reaction was characterized by a coefficient of variation of 41%, above the generally accepted 35% threshold (Klymus et al., 2020), with the next highest standard (500 copies per reaction) characterized by a coefficient of variation of 17%. The LOD for detection in a single well is 5 copies per reaction (Ct of 39.4), defined as the lowest concentration in which at least 95% of the wells were positive. Dose–response modeling of LOD was not solvable because there was insufficient variation in the results of the LOD experiment: only one of the tested concentrations was detectable in some, but not all, of the replicates tested (Klymus et al., 2020). Higher-resolution testing such that multiple concentrations were detectable in some, but not all, would be needed for the dose–response modeling to be solvable. Notably, mpox was detected in 8 of 10 (80%) replicates with 3 copies per reaction (the theoretical lower limit for qPCR) with a mean Ct of 40.4, corresponding to a 96% likelihood of detection in at least one of the duplicate wells. No mpox DNA was detected in 56 NTC wells or in the 46 wells from the 23 samples processed from March to April 2022, before the first reported mpox case. Specifically, no NTCs or negative control samples showed exponentially increasing fluorescence above the threshold.

### Mpox DNA Concentration and Loads in Wastewater

Of the 365 samples analyzed between 9 March and 31 October 2022, 359 (98%) were analyzed in duplicate and 6 (2%) in singlet. To compare the data obtained from the clinical cases, positive samples were defined by both the definition of lower LOD as 95% likelihood of detecting a sample and by the definition of exponentially-increasing fluorescence. The LOD definition based on dose–response modeling was not solvable as described, so was not further pursued. Using the definition of LOD as 95% likelihood of detection in a sample, only 5 (1%) of the samples were positive as defined by detection of at least 5 copies per reaction (Ct lower than 39.4) in at least one replicate (Figure S2). Using the definition of exponentially increasing fluorescence above the threshold, 79 (22%) samples had detectable mpox DNA in at least one replicate. As the 45^th^ cycle was the terminal QPCR cycle used in the protocol, this definition is approximately equivalent to 0.12 copies per reaction based on extrapolation of the standard curve. Notably, this concentration would not be reliably detected because QPCR technology requires at least one complete copy of the target region; detection at high cycle numbers is therefore stochastic. Notably, in 19 samples (24% of positive samples), both replicate wells tested positive with this definition.

The average (standard deviation) Ct of all positive wells was 41.1 (1.2). Extrapolating these wells to concentrations results in a mean (standard deviation) of 2.0 (1.4) gce per reaction, or 0.8 (0.6) gce per ml wastewater (Fig. 1B). Corresponding arithmetic mean (standard deviation) loads were 1 × 10^11^ (9 × 10^10^) gce per day (Fig. 1C). Given all measured concentrations were well below the LOQ, further analyses were conducted only on the detect/non-detect data.

### Sensitivity, Specificity, and Accuracy of Wastewater-Based Detection Relative to Reported Clinical Cases

Each of the three cantons reported cases for 23 calendar weeks from 16 May to 23 October, for a set of 69 weeks with corresponding number of clinically reported cases (Fig. 1A). Additionally, wastewater samples from all three sites before (March and April) were tested one time per week when no clinical cases were reported, for a set of 30 calendar weeks across three sites.

Alignment of detection with cases was performed based on the different definitions. Because one (dose–response modeling) was not solvable, further analysis was not possible. Using the standard definition of LOD as detection of a positive well with 95% likelihood, mpox DNA was detected in 5 calendar week-cantons, of which 4 occurred coincident to reported cases in the canton. Although specificity (98%) was high, sensitivity (10%) and accuracy (56%) were low (Table 2). Using the more liberal definition of LOD based on exponentially increasing fluorescence above the threshold, mpox DNA was detected in 27 calendar week-cantons with reported cases. Although specificity (90%) was reduced, it remained high. Both sensitivity (64%) and accuracy (78%) increased appreciably (Table 2). Notably, no wastewater detections occurred in the sampling period before and after the reported cases (Fig. 1B).

**Table 2:** Confusion matrices of mpox DNA detection within a calendar week at each catchment relative to reported clinical cases in the corresponding canton in the coincident calendar week

	One or more reported cases in the calendar week	No reported cases in the calendar week
LOD defined by minimum Ct with 95% likelihood of detection		
Wastewater Detect	4	1
Wastewater Non-Detect	38	47
*Sensitivity: 10%; Specificity: 98%; Accuracy: 56%*		
LOD defined by any qPCR fluorescence		
Wastewater Detect	27	5
Wastewater Non-Detect	15	43
*Sensitivity: 64%; Specificity: 90%; Accuracy: 78%*		

Detection of mpox defined as minimum Ct with 95% likelihood of detection or defined as any exponentially increasing qPCR fluorescence above the threshold. Detection of mpox based on dose–response modeling following Klymus et al. (2020) was not solvable and therefore not analyzed (Ye et al., 2016)

Alternative definitions of the LOD, such as LOD as detection of a positive well with 50% likelihood or LOD50, are also possible and would impact both sensitivity and specificity of the assay. Given that the detection of mpox DNA in a single well was overwhelmingly associated with reported clinical cases of mpox in the same calendar week, increasing the Cq threshold used to define limit of detection would increase sensitivity while reducing specificity slightly (Figure S2).

### Impact of Reported Clinical Cases on Likelihood of Mpox Detection

**Fig. 2:**
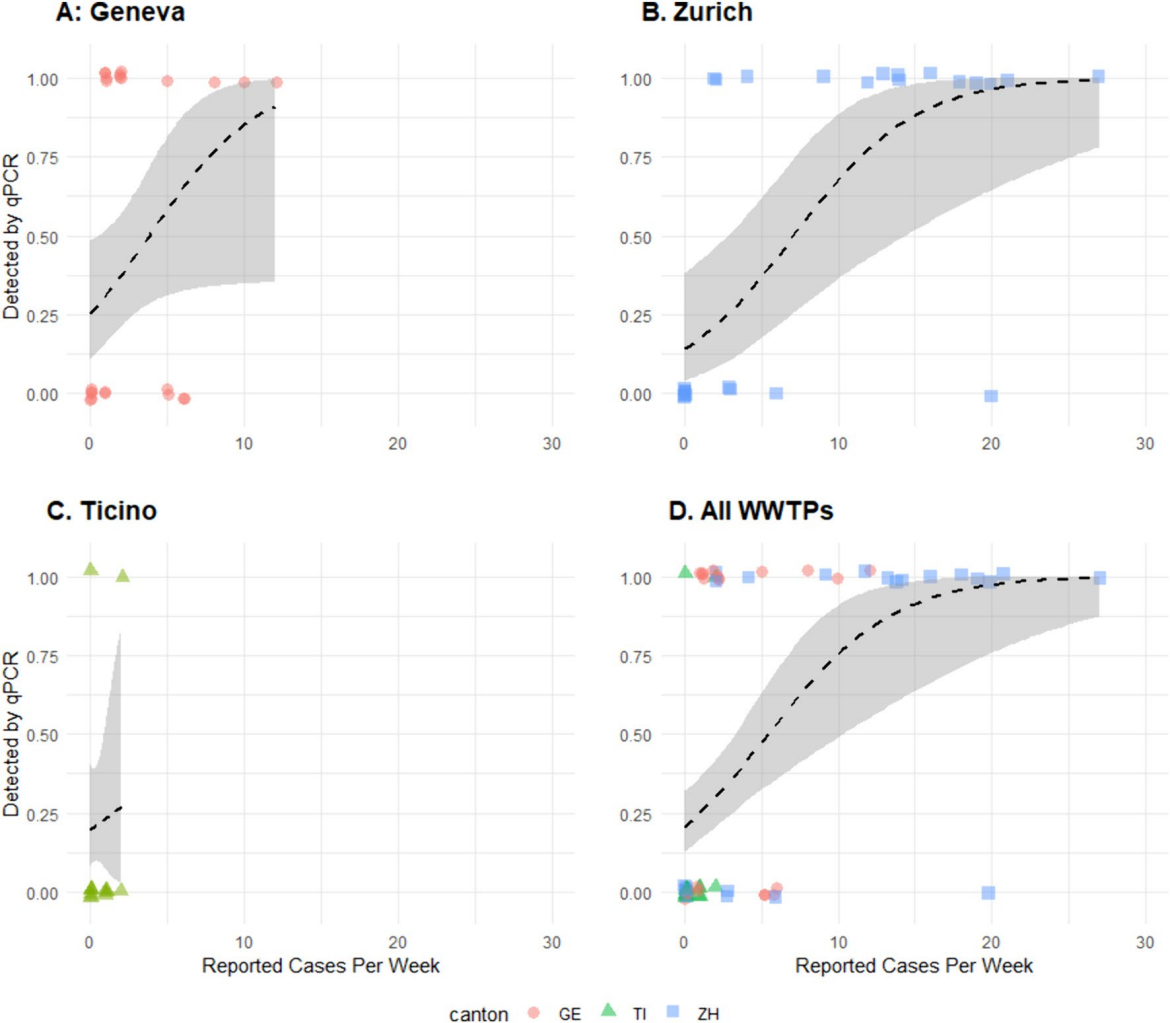
Logit regression for detection of mpox DNA in one or more samples during a calendar week as a function of the reported cases within the same calendar week for **A** STEP Aïre in canton Geneva (GE), **B** ARA Werdhölzli in canton Zurich (ZH), **C** CDA Lugano in canton Ticino (TI), and **D** all wastewater treatment plants. Detection, here, is defined as any exponentially-increasing qPCR fluorescence above the threshold. Dashed lines represent fit logistic regression models with shaded areas representing the 95% confidence interval

The likelihood of detection of mpox DNA in one or more replicate wells per week, based on the definition of exponentially increasing fluorescence above the threshold, increased with increasing number of reported clinical cases across all three sites (Fig. 2). When aggregating data across all three sites, there was a 50% likelihood of detecting 5.5 cases [95% CI 3.2, 10.3] (Fig. 2). Correspondingly, the average likelihood of detecting mpox DNA when there was at least one reported clinical case in the canton was 25% [95% CI 16%, 36%], which rose to 75% [41%, 91%] when there were 10 or more cases (Table 3). Mpox DNA was detected in the wastewater within all catchments when there were more than 6 reported clinical cases within the catchment with one exception: 20 reported cases in Zurich, which occurred in early July (Figs. 1 and 2D).

**Table 3:** Estimated likelihood of detection in one or more qPCR samples or replicates and corresponding 95% confidence interval of 1, 5, 10, and 20 reported clinical cases within a catchment within a calendar week

Reported cases	Likelihood of detection [95% CI]
1	25% [16%, 36%]
5	47% [32%, 63%]
10	75% [41%, 91%]
20	97% [75%, >99%]

## Discussion

Mpox DNA was detectable in raw wastewater influent of three treatment plants in Switzerland coincident to the outbreak in 2022, in line with previous reports (Jonge et al., 2022; La Rosa et al., 2023; Sharkey et al., 2022; Tiwari et al., 2023; Wolfe et al., 2023). All detections were at concentrations below the LOQ, and most were below the lowest quantity reliably detected, defined as detectable >95% of the time in singlet. Further, many detections were characterized by inconsistent replicates in which signal was detected in only one of the two replicates. Despite the uncertainty in the data, wastewater-based detection of trace concentrations of mpox DNA aligned with clinically reported cases, showing high accuracy, sensitivity, and specificity within a calendar week. The analyses, based on uncertain and variable wastewater and clinical data, highlight the potential utility of wastewater-based surveillance to provide insights into the epidemiology of an emergent disease occurring at relatively low-incidence rate.

### Detection of Mpox DNA in Wastewater Coincident to Reported Clinical Cases was Observed Despite Very Low Concentrations and Inconsistency in Replicates

Detection of pathogens in wastewater using molecular methods is challenging due to variation in shedding prevalence rates, intermittency in shedding into wastewater, substantial dilution and loss during transport, low methodological recovery rates, and co-concentration of inhibitory substances. All of these factors compound to result in intermittent and variable detection when infection prevalence is low and there are correspondingly few contributing shedders. The low concentrations observed here (mean of 2 gce per reaction) are estimated based on extrapolation below the theoretical lower limit of 3 gce per PCR reaction (Bustin et al., 2009). Furthermore, the majority of detections occurred in only one of two replicate wells. Although the lower LOD is defined as the lowest concentration at which 95% of positive samples can be reliably detected, in complex environmental samples with targets at concentrations so low that detection becomes stochastic, it may be useful to use an alternative definition. As shown, using a definition of exponentially increasing fluorescence above the threshold may still lead to meaningful data, especially when no similar signal is observed in NTCs and other negative controls. The specificity in our assay provides confidence that detects, even intermittent and at low concentrations, likely reflect shedding of mpox DNA by an infected person into wastewater.

### Alignment of Wastewater-Based Detection of an Emergent Pathogen at Low Incidence with Clinical Case Reporting is Influenced by the Sensitivity of the Method for Detection and Quantification in Wastewater

Insufficient assay sensitivity may lead to the failure to detect DNA in wastewater despite circulating clinical cases. In our data set, we observed fifteen calendar weeks in which cases were reported despite no detection of signal in the wastewater. Nine of the fifteen non-detects occurred in weeks with 3 or fewer reported cases. The lack of detection of mpox DNA could occur for a number of reasons. For example, infected people may not shed sufficient amounts of DNA into the wastewater. Indeed, in a study of 12 people infected with mpox, 4 (33%) people did not shed detectable DNA in the 1–3 fecal samples tested, and 3 (25%) people did not shed in the 1–3 urine samples tested (Pieró Mestres et al., 2022). Additionally, sample inhibition or insufficient method sensitivity may lead to the failure to detect mpox DNA in the wastewater. Previous studies reporting mpox detection in wastewater solids, as opposed to the influent used in this study, may benefit from increased sensitivity due to partitioning of enveloped viruses in solids (Sharkey et al., 2022; Wolfe et al., 2023; Ye et al., 2016). The absence of wastewater-based detection of a pathogen therefore does not imply the absence of infections.

### Specificity of Methods Influences Confidence in Wastewater-Based Surveillance Results

Wastewater-based surveillance approaches are reliant on molecular methods typically developed for clinical samples. These methods may suffer from lack of specificity, particularly in the complex matrix of wastewater. Lack of specificity may lead to detections in the absence of infections. These “false positives” pose risks—such as an inappropriate shifting of resources—if reported to stakeholders. To mitigate false positives, detections can be confirmed using alternative methods, such as sequencing (i.e., amplicon sequencing) (Boehm et al., 2023); using multiple, distinct, or confirmatory assays (Wolfe et al., 2023); or demonstrating non-detects in archived samples prior to an outbreak.

Sequencing is likely the gold standard for a confirmatory assay, particularly if the genomic region sequenced is specific to the target pathogen. Positive confirmatory assays targeting multiple genomic regions unique to the target also provide a high degree of certainty. Less reliable, but still valuable, is testing of archived samples. Failure to detect a pathogen in samples outside of the known period of circulating infections provides some confidence that the assay is specific to the disease. However, this approach relies on the assumption that lack of specificity of the assay would be detected by processing archived samples. That is, causes of false positive detection occur at a sufficient frequency and concentration in archived samples that they are detected. An additional limitation of relying on archived samples is that if the target pathogen is detected in archived samples, it does not provide any clarity on the assay specificity. Although one interpretation is that the assay lacks specificity, another interpretation is that the target pathogen circulated in the community undetected by clinical surveillance. In this case, additional investments in confirmatory assays or sequencing approaches are recommended. In this study, we investigated archived samples. Piloted attempts to sequence gel-purified amplicons of the G2R gene using Sanger sequencing based on same primers used for testing were not successful, potentially due to an insufficient concentration of purified DNA. Nested PCR prior to sequencing, as conducted by Wolfe et al., (2022) for influenza confirmation in wastewater (Wolfe et al., 2022), may be more successful for the low observed target concentrations, but requires additional assay design and optimization. Nested sequencing also risks potential cross-contamination (Marmirol et al., 2007). Specificity testing based on archived samples requires robust and accurate clinical surveillance on the emergence of a disease into the catchment; insufficient clinical surveillance can otherwise lead to detection in wastewater inappropriately attributed to insufficient assay specificity.

Confirmation methods improve data quality and reliability, but delay sharing of information obtained from wastewater to stakeholders, and subsequently delay any associated public health actions. The ethical guidelines of wastewater-based surveillance call for both assuring high quality, reliable data as well as effectively reporting in a timely manner (Hrudey et al., 2021). For emergent diseases requiring novel assays, information sharing with stakeholders could couple early results with clear descriptions of methodological uncertainty (Maxim and van der Sluijs 2011).

### Uncertainty in Reported Clinical Case Data Confounds Assessment of Wastewater for Surveillance

The alignment observed here between reported clinical cases and wastewater-based data assume reported clinical cases is an accurate indicator of both magnitude and timing of infections in the community. In reality, reported clinical cases likely represent only a portion of true cases, and only a portion of infections shed DNA into wastewater. Furthermore, the timing of clinical case reporting may be shifted from the timing of DNA shedding. Detection of DNA in wastewater can therefore occur despite an absence of reported clinical cases for a number of reasons: 1) reported clinical cases likely represent only a portion of the true number of infections, 2) infected people shed for weeks after symptom onset (Pieró-Mestres et al., 2022), and there may be misalignment in the timing of a reported case and shedding, and 3) people are mobile between catchments and may shed in different cantons to which they were tested. In the data set, five weeks were associated with detections in the absence of reported cases, all within CDA Lugano in Ticino, which reported the lowest incidence rate of the three catchments. Improved clinical surveillance, and surveys to understand mobility of cases within and between wastewater catchment areas, could improve understanding of alignment between cases and wastewater data.

Our findings support a recommendation for the use of a liberal definition of the limit of detection in wastewater-based surveillance programs, particularly for emergent diseases circulating at low prevalence. Timely access to this data may help inform rapid responses and resource allocation during a public health crisis. However, we note that liberal definitions are potentially associated with higher risks of false positives; this risk should be clearly communicated to public health officials for consideration in their decision making process. Reevaluation of the definition of the lower of the detection is nevertheless recommended, for example: 1) after an assay is established and sensitivity and specificity have been assessed; 2) if the threat of disease spread is low, or the disease is already endemic; or 3) if clinical surveillance is deemed sufficiently robust to warrant reduced reliance on detection based on wastewater surveillance.

## Conclusions

Mpox DNA was detectable in the wastewater influent from three treatment plants in Switzerland, with the probability of detection statistically significantly linked with the number of reported clinical cases at the cantonal level. Wastewater-based surveillance approaches may provide rapid insights into the extent (geographic distribution, magnitude of disease burden) of emerging pathogens, even those intermittently detected at low concentrations. Increasing the timeliness of the response of wastewater-based surveillance to an emergent pathogen would benefit from further early investments in prioritizing surveillance as a complementary approach to clinical surveillance; development of assays and archiving associated molecular material (primers, probes, positive controls) for potential threats; and recognizing uncertainties associated with detection and risks of false positives.

## Supplementary Information

Below is the link to the electronic supplementary material.

Supplementary file1 (DOCX 48 KB)

## Footnotes

1 https://www.gov.uk/government/news/expansion-of-polio-sewage-surveillance-to-areas-outside-london (accessed 28.11.2022).

2 https://www.bag.admin.ch/bag/en/home/krankheiten/ausbrueche-epidemien-pandemien/aktuelle-ausbrueche-epidemien/affenpocken/situation-einschaetzung.html (accessed 28.11.2022).

3 https://www.who.int/news/item/23-07-2022-second-meeting-of-the-international-health-regulations-(2005)-(ihr)-emergency-committee-regarding-the-multi-country-outbreak-of-monkeypox

4 https://www.paho.org/en/file/109251/download?token=kCLnmgNR#:~:text=Laboratory%20Diagnosis%20of%20Monkeypox,-3&text=The%20recommended%20specimen%20type%20for,is%20skin%20lesion%20material%2C%20including%3A&text=Swabs%20of%20lesion%20surface%20and,mixed%20in%20the%20same%20tube (accessed 28.11.2022).
