# Supplementary material for: Monitoring an Emergent Pathogen at Low Incidence in Wastewater Using qPCR: Mpox in Switzerland
Source: Food Environ Virol. 2024 May 23;16(3):269–79. doi: 10.1007/s12560-024-09603-5 (PMC11422434; doi:10.1007/s12560-024-09603-5)
Supplement: Supplementary file 1 — Supplementary file1 (DOCX 48 KB) [file 12560_2024_9603_MOESM1_ESM.docx]

**Supplementary Files for: Monitoring an Emergent Pathogen at Low Incidence in Wastewater using qPCR: Mpox in Switzerland**

**Authors:** Timothy R. Julian^1,2,3,*^, A. J. Devaux^1^, Laura Brülisauer^1^, Sheena Conforti^1,4^, Johannes C. Rusch^1^, Charlie Gan^1^, Claudia Bagutti^5^, Tanja Stadler^4,6^, Tamar Kohn^7^, and Christoph Ort^1^

**Affiliations:**

^1^Eawag, Swiss Federal Institute of Aquatic Science and Technology, Ueberlandstrasse 133, Dübendorf CH-8600, Switzerland

^2^Swiss Tropical and Public Health Institute, Allschwil CH-4123, Switzerland

^3^University of Basel, Basel CH-4001, Switzerland

^4^Department of Biosystems Science and Engineering, ETH Zurich, Basel CH-4058, Switzerland

^5^State Laboratory of Basel-Stadt, Basel, Switzerland

^6^Swiss Institute of Bioinformatics, Lausanne CH-1015, Switzerland

^7^Laboratory of Environmental Chemistry, School of Architecture, Civil and Environmental Engineering, (ENAC), École Polytechnique Fédérale de Lausanne, Lausanne, Switzerland

**Corresponding Author:** Timothy R. Julian, [tim.julian@eawag.ch](mailto:tim.julian@eawag.ch), +41 58 765 5632


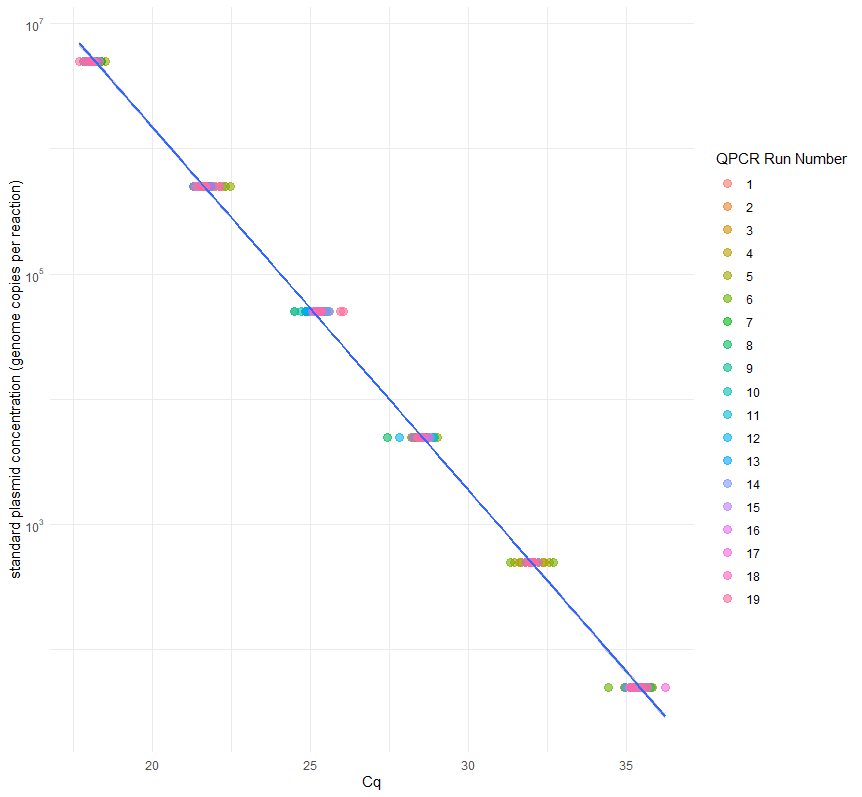


**Figure S1**: The Cq of the standard curves of 50 to 5*10^6^ genome copies per reaction of the standard plasmid across all QPCR runs.


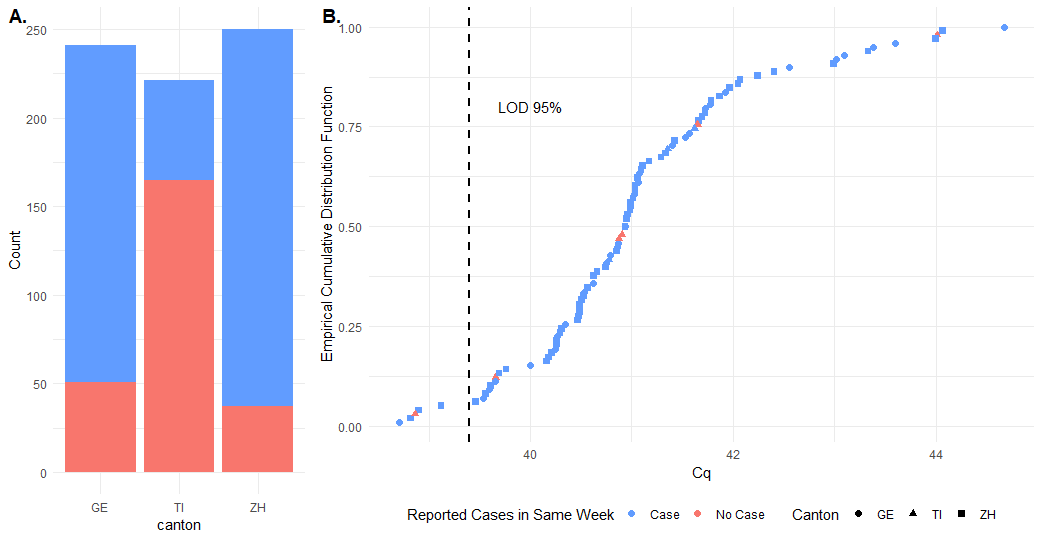


**Figure S2:** A: Count of QPCR sample wells without detectable mpox DNA by canton colored by whether there was a clinical mpox case reported in the canton during the same calendar week (blue) or not (red). B: Empirical cumulative distribution function of the Cq values of positive QPCR sample wells with detectable mpox DNA colored by whether there was a clinical mpox case reported in the canton during the same calendar week (blue) or not (red). The vertical line at a Cq of 39.4 represents the threshold based on the 95% confidence of detection. A Cq of 45 is the terminal cycle.
